# Curcumin Derivative Cur20 Attenuated Cerebral Ischemic Injury by Antioxidant Effect and HIF-1α/VEGF/TFEB-Activated Angiogenesis

**DOI:** 10.3389/fphar.2021.648107

**Published:** 2021-04-15

**Authors:** Runfang Zhang, Tingkui Zhao, Beibei Zheng, Yun Zhang, Xiaohui Li, Feng Zhang, Juan Cen, Shaofeng Duan

**Affiliations:** ^1^ Key Laboratory of Natural Medicine and Immune Engineering, School of Pharmacy, Henan University, Kaifeng, China; ^2^ Biology Institute, Qilu University of Technology (Shandong Academy of Sciences), Jinan, China; ^3^ Department of Neurology, The First Affiliated Hospital of Henan University, Kaifeng, China; ^4^ Henan International Joint Laboratory of Chinese Medicine Efficacy, Henan University, Kaifeng, China

**Keywords:** curcumin derivative, vascular dementia, brain microvessel endothelial cells, angiogenesis, zebrafish

## Abstract

In this paper, a curcumin derivative Cur20 was synthesized for better hydrolytic stability, which showed a higher angiogenic effect on zebrafish model than curcumin. In order to reveal the potential effects on neuroprotection, a mouse model of vascular dementia (VaD) induced by permanent right common carotid artery occlusion (rUCCAO) was established. After two weeks of curcumin administration, the cognitive function of mice was detected by Morris water maze and Y maze. The alteration on oxidative injuries and morphological damage were also analyzed by reactive oxygen species, superoxide dismutase, GSH, malondialdehyde tests, and Nissl stain on cortex/hippocampus. The angiogenesis and related signal factors were evaluated as well. The results showed that Cur20 significantly attenuated the cognitive dysfunction and histopathological changes of the VaD mice with enhanced antioxidant system and angiogenesis. In addition, primary rat brain microvessel endothelial cells (rBMECs) with oxygen glucose deprivation (OGD) were applied to further verify the possible mechanisms of Cur20-induced angiogenesis. The results demonstrated that the proliferation effect and the activation of pro-angiogenesis factors such as HIF-1α, VEGF, and TFEB might contribute to the protection of ischemic injury. Based on the above, our conclusion is that Cur20 can be considered as a promising therapeutic strategy for VaD.

## Introduction

With the aging of the population and the change of dietary structure, the incidence of cardiovascular and cerebrovascular diseases increases year by year, thus leading to a gradual increase of dementia patients. Dementia is a syndrome of developmental disability that involves varying degrees of cognitive, emotional, language and consciousness disorders. Vascular dementia is the second most common type of dementia after Alzheimer’s disease (AD) (Farooq et al., 2017). Extensive clinical evidence suggests that chronic cerebral perfusion is associated with cognitive decline and hippocampal neuron damage in neurodegenerative diseases (Sinha et al., 2020). Therefore, VaD, which is usually caused by multiple strokes or long-term chronic cerebral ischemia, accompanied by a gradual decline in memory, emotion control and cognitive function. It seriously damages the health of patients, affects the quality of life of patients, and imposes a heavy burden on society and families. However, there has been no FDA approved treatment to control the progress of this disease to date (Venkat et al., 2015). Drugs that dilate blood vessels and increase cerebral blood flow, or ant-inflammation drugs or free radical scavengers are often used in clinical treatment. However, only donepezil and galantamine has shown some efficacy in AD/VaD. The beneficial effects of other drugs, such as rivastigmine, memantine, nimodipine, piracetam, and herbal therapies, such as citicoline, actovegin, huperzine A, and vinpocetine, have not been established (Farooq et al., 2017). However, traditional Chinese medicines such as ginkgo biloba, ergeron and acanopacanthus are difficult to meet clinical needs despite their promising reports due to their unclear active ingredients, unclear mechanism of action, difficult quality control and large side effects (Shuyun et al., 2019). Hence, there is an urgent need to develop more effective drugs to treat this disease (Chan et al., 2018).

Curcumin, the most active compound in Curcuma, is widely used in India and other Asian countries, not only as a flavor, but also as an important drug (Xu et al., 2019). The clinical application of flavonoids is related to their structural activities such as antioxidant, anti-inflammatory, anti-parasitic, anti-mutagenesis, chemical protection, antiviral and tumor prevention (Yunfeng et al., 2017). However, the quality of curcumin is difficult to control because of its instability and easy degradation. Although the stability of curcumin can be improved by adding demethoxycurcumin or sodium dodecyl sulfate, the use of additives not only increases the production cost, but also brings various unsafe risks (Dey and Sreenivasan, 2014). Therefore, our team synthesized a series of curcumin derivatives by modifying the unstable part of curcumin (Feng et al., 2014). In addition to the reported compounds, this study also confirmed that the stability of Cur20 was significantly improved without affecting its good antioxidant activity. Since curcumin has been reported to have neuroprotective effects due to its antioxidant (Mhillaj et al., 2019), we are curious whether Cur20 has its own mechanism of potential activity against VaD.

In this study, zebrafish was first utilized to screen the potential angiogenic activity of Cur20. Then, *in vivo* and *in vitro* VaD models were applied to evaluate the exact efficacy and the mechanism of Cur20. As an important type of vascular dementia, subcortical ischemic vascular dementia is usually produced in animal models, such as bilateral permanent common carotid artery occlusion (2-VO) in rats and right common carotid artery occlusion in mice (Yao et al., 2019). In view of the characteristics of 2-VO experimental animals, such as high mortality, serious injury of motor system, and difficult detection of behavioral indicators, we chose the rUCCAO model with simple operation, low mortality, good repeatability and little trauma. Then, the effects of Cur20 on rUCCAO mice were evaluated at molecular, histological and behavioral levels. In addition, the oxygen and glucose deprivation model of primary rat brain microvascular endothelial cells (RBMECs) was used to further reveal the mechanism of Cur20.

## Materials and Methods

### Hydrolytic Stability Test

**FIGURE 1:**
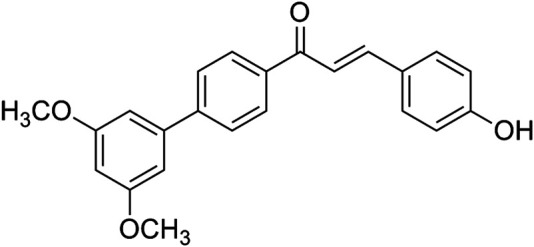
The structure of Cur20 (1—(3′, 5′- dimethoxy—[1, 1′—yl) biphenyl]—4–3—(4—hydroxyphenyl) prop—2—en—1—one).

The structure of Cur20 was shown as Figure 1, and the synthesis process was presented in Supplementary Material. HPLC was used to verify the hydrolytic stability of curcumin and Cur20 (Feng et al., 2014). Briefly, the compound was dissolved in DMSO to prepare a stock solution. Then, three solutions (A, B and C) were prepared for each drug as follows: A, dilute stock solution with anhydrous ethanol to prepare 20 μM solution as the control; B/C, dilute stock solution with 50% anhydrous ethanol and 50% phosphoric acid buffer solution (pH 7.4) to form 20 μM solution. In the experiment, Solution C should be prepared first and placed in a cell culture chamber at 37°C for 48 h. Before the sample was taken out, Solution A and B were prepared, and all samples were determined by HPLC (Agilent 1,100) with Diamonsil C18 chromatographic column (250mm × 4.6 mm, 5 microns). The mobile phase was methanol-isopropanol-water-glacial acetic acid (20:27:48:5); the flow rate was 0.5 ml/min; the column temperature was 25°C; the injection volume was 20 μL; and the detection wavelengths of curcumin and Cur20 were 426 and 356 nm, respectively. Degradation rate = (peak area of group B solution—peak area of group C solution)/peak area of group B solution.

### Zebrafish Intersegmental Vessel (ISV) Angiogenesis Assay

Transgenic zebrafish with vascular-specific fluorescence Tg (Flk-1:GFP) were used, and all experiments were conducted according to the standard ethical guidelines and under control of Biology Institute, Qilu University of Technology of committee, and were in accordance with the National Institutes of Health’s Guide for the Care and Use of Laboratory Animals. After fertilized eggs were disinfected and washed, they were transferred to zebrafish embryo culture water at 28°C with controlled light. After fertilized eggs developed for 20 h post-fertilization (hpf), normal developing zebrafish larvae were selected, placed in 24-well plates (8 zebrafish larvae/well) and treated as follows: blank control group (embryo culture water), model group of PTK787 (Sigma, 0.2 μg/ml), curcumin (Sigma, 50 μM curcumin + 0.2 μg/ml PTK787) and Cur20 group (50 μM Cur20 + 0.2 μg/ml PTK787). After the drugs were incubated for 24 h, (i.e. 44 hpf), the effect of drugs on ISVs length of zebrafish was observed and calculated under a fluorescence microscope with Image Pro Plus software (Chen et al., 2018).

### Vascular Dementia Mouse Model

**FIGURE 2:**
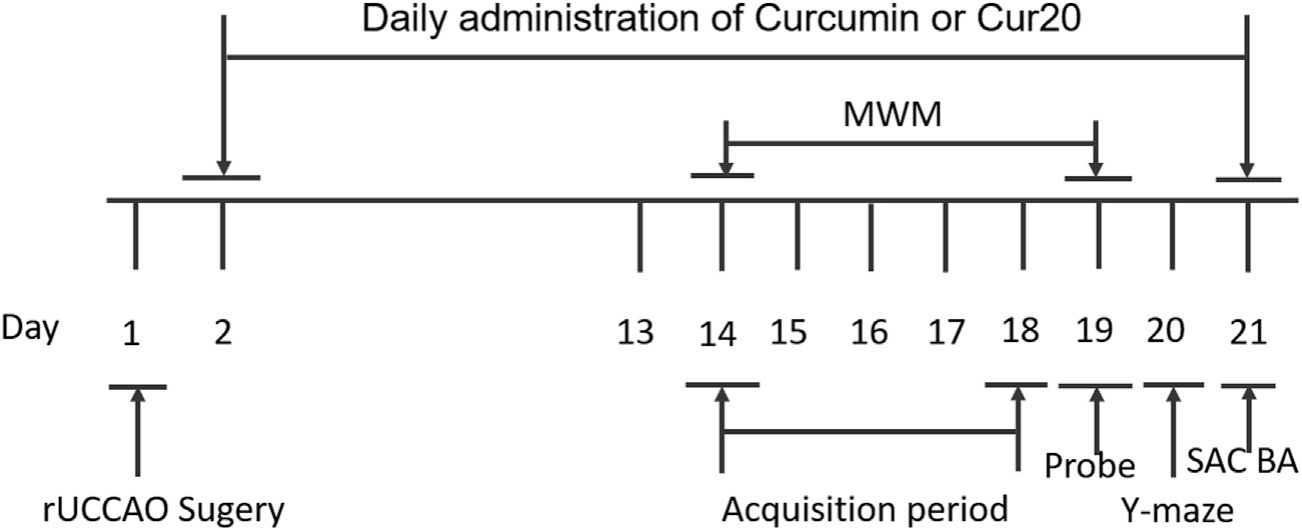
Experimental schedule for *in vivo* experiment. The mice were randomly divided into four groups: sham control group; VaD model group; curcumin group; and Cur20 group. rUCCAO, right unilateral common carotid arteries occlusion; MWM, Morris water maze; SAC, sacrifice; BA, biochemical analysis.

Male ICR mice (20–25 g, 6 weeks old) were purchased from the laboratory animal center of Henan Province (Zhengzhou, China) and maintained under specific pathogen free (SPF) conditions. All experimental protocols were performed according to Principles of Laboratory Animal Care and approved by the Animal Ethics Committee of Henan University (Kaifeng, China), and were in accordance with the National Institutes of Health’s Guide for the Care and Use of Laboratory Animals. The mice with permanent right common carotid artery occlusion was used as a VaD model, as previously described (Liu et al., 2015). The mice were anesthetized and fixed on an operating table. The right common carotidartery was isolated and sutured permanently with a small diameter silk thread. The rectal temperature was maintained at 37 ± 0.5°C with a homoeothermic pad controlled during the VaD surgical procedure. A 30% drop in the regional cerebral blood flow was considered as a successful VaD mouse model, which was measured by laser-Doppler fluxmetry (LDF) (MP150 starter system, BIOPAC system, Inc., United States). The mice were randomly divided into four groups: sham control group (*n* = 6); VaD model group (*n* = 6), which was subjected to rUCCAO; curcumin group (*n* = 6), which was subjected to rUCCAO and treated with 20 μmol/kg curcumin; and Cur20 group (*n* = 6), which was subjected to rUCCAO and treated with 20 μmol/kg Cur20. All animals were treated intragastrically with physiological saline or drugs following rUCCAO once a day for 2 weeks. Then behavioral tests involving Morris water maze and Y maze were utilized. During the behavioral evaluation, drugs were administered after the tests every day. At the end of the experiment, the mice were euthanized and their brain samples were collected for analysis (Figure 2).

### Morris Water Maze

After two week of drug treatment, the learning and memory skills of animals were confirmed in the Morris water maze (MWM, ZS-001, Zhongshidichuang Technology Development Co., Ltd.). In the training experiment, the mice were placed in water at four different starting quadrant points from day 1 to day 5 and trained to find the submerged platform within 60 s. Each mouse was allowed to stay on the platform for 5 s prior to being returned to its home cage. If a mouse was unable to locate the platform within 60 s, it was gently guided to the platform and allowed to stay there for 15 s. The time spent finding the platform is termed as the escape latency. On the sixth day, the platform was removed from the tank and each mouse was tested in a probe trial in which the time spent in the former platform-containing quadrant was recorded. The number of platform crossings was recorded during this probe trial as well.

### Y-Maze

The Y-maze test relies on the innate tendency of rats to explore a novel environment. The apparatus consists of three arms, which was labeled as A, B, and C. Briefly, at 1 h post the last drug administration, the animals were subjected to the Y-maze task. The mice were placed in the center of the Y-maze facing the south arm B and were allowed to explore the maze freely for a period of 5 min. The number and the sequence of arm entries were recorded by an observed unaware of the treatment groups. Alternation behavior was defined as consecutive entries into all three arms, (i.e., ABC, CAB, or BCA but not BAB) (Sarter et al., 1988). The percentage of spontaneous alternation was measured as an index of working memory = [(number of alternations)/(total number of arm entries—2)] × 100. The total number of arm entries was recorded as an index of locomotor activity.

### Nissl Staining

The brains of the mice were fixed in 4% paraformaldehyde, dehydrated by 100% ethanol and then immersed with butanol. Then, the slices were stained with Cresyl Violet acetate and glacial acetic acid (Sigma, St. Louis, MO, United States). After that, they were dehydrated and covered with coverslip. To count the number of viable neurons, four random fields of vision in each slice were measured per mouse using light microscope (Leica TCS SP8, Germany) at ×200 magnification.

### Immunohistochemistry (IHC)

Brain slices were incubated with antibodies (BosterBio-engineering, Wuhan, China) against HIF-1α, CD34, NF-κB, VEGF, TFEB (rabbit polyclonal IgGantibody; 1:100) at 4°C and reacted with avidin-biotin-peroxidase complex and DAB (Boster Bio-engineering, Wuhan, China). The levels of these proteins in ischemic hemisphere were investigated using light microscope at a magnification of ×200. The positive cells were determined as mean the gray value.

### Biochemical Assays

After behavior tests, the mice were anesthetized with chloral hydrate and rapidly decapitated. The skull was cut open and the brain was exposed from its dorsal side. The whole brain was quickly removed and homogenized in a 0.03 M sodium phosphate buffer with a pH of 7.4 employing a homogenizer. The homogenate was used for the determination of reactive oxygen species (ROS), malondialdehyde (MDA), glutathione (GSH), and superoxide dismutase (SOD) by assay kits of Beyontime Institute of Biotechnology (Shanghai, China). DCFH-DA was utilized to evaluate the level of cerebral ROS level (Heimfarth et al., 2013). MDA, an indicator of lipid peroxidation, was spectrophotometrically measured using the thiobarbituric acid assay procedure as previously described by Ohkawa et al. (Ohkawa et al., 1979). GSH, the endogenous antioxidant, was determined by its reaction with 5, 5′-dithiobis (2-nitrobenzoic acid) (Ellman’s reagent) to yield a yellow chromophore which was measured spectrophotometrically. The activity of SOD was assayed according to the method described by Winterbournet al. (Winterbourn et al., 2002).

### Primary Culture of Rat Brain Microvessel Endothelial Cells (rBMECs) and Oxygen-Glucose Deprivation

The rBMECs were isolated according to the previous method (Ji et al., 2013). Cells were cultured in DMEM/F12 (1:1) medium supplemented with 20% fetal bovine serum at 37°C in 5% CO_2_ humidified atmosphere. The cell viability of the rBMECs was determined via MTT assay after Cur20 treatment (0.1, 1, 10, 25, 50 μM) for 48 h. OGD was performed in rBMECs cells according to the described method (Ji et al., 2013). Cells were pretreated with 2 mM NAC or 0.1, 1, 10 μM Cur20 for 2 h before exposure to OGD. Then, the neuroprotection effect of Cur20 was also detected by MTT assay.

### Flow Cytometry Determination of Apoptosis and Reactive Oxygen Species

The Annexin V-FITC/PI apoptosis assay kit and ROS assay kit were purchased from Key GEN Biological Engineering Materials Co., Ltd. (Nanjing, China). Briefly, the cell suspension was centrifuged and re-suspended in 195 μL Annexin V-FITC binding buffer and incubated with 5 μL Annexin V-FITC in the dark at ambient temperature for 10 min. The cells were then centrifuged, and the pellet was re-suspended in 195 μL binding buffer. The cells were then incubated with 10 μL PI solution on an ice bath in the dark for 10 min. The suspension of each group was analyzed by Flow Cytometry (BD FACSVerse). For ROS assay, the cells were seeded on 6-well plates. After incubation with various chemicals, the cells from each group at a density of 1 × 10^6^ were harvested and incubated with 10 μM DCFH-DA at 37°C for 30 min in the dark. Then, the cells were washed with PBS, and the fluorescence intensity was measured for ROS with Flow Cytometry (BD FACSVerse).

### Immunofluorescence

The expression of Ki67 and the expression and location of TFEB were detected by immunofluorescence to observe the alteration after drug treatment. Cells were grown on glass coverslips, and then fixed in 4% paraformaldehyde in PBS for 20 min after drug treatment. Then, the cells were washed twice with ice cold PBS and incubated for 10 min with PBS containing 0.25% Triton X-100. After being blocked with 5% BSA for 30 min, the cells were incubated with primary antibody in 0.5% BSA/PBST in a humidified chamber overnight at 4C. Subsequently,the cells were washed with TBST for three times and incubated with FITC-conjugated secondary antibody at room temperature for 1 h, followed by incubation with 10 μg/ml DAPI for another 30 min. Signals were visualized and recorded using a Confocal Microscopy (Leica TCS SP8).

### Western Blot Analysis

Proteins were collected and their concentrations were determined with a BCA protein detection kit (Beyotime Institute of Biotechnology, Shanghai, China). The samples (20 μg) were separated using sodium dodecyl sulfate polyacrylamide gel electrophoresis (SDS-PAGE) and transferred onto a nitrocellulose membrane (Bio-Rad, Hercules, CA). The membranes were incubated in blocking solution containing 5% nonfat milk in TBST at room temperature for 1.5 h and then incubated with the primary antibodies diluted in the blocking solution individually overnight. Then, the membranes were incubated with HRP conjugated secondary antibodies (Boster Bio-engineering, Wuhan, China) for 1 h at room temperature. The bound antibodies were detected using a chemiluminescence system (ECL Plus, Thermo Scientific, and Rockford, IL).

### Statistical Analysis

All the experiments were performed in triplicates. All the data were presented as means (±S.D.). Significant differences between the groups were determined by One-way ANOVA followed by Dunnett’s multiple comparison tests. *p*-values less than 0.05 were considered as statistically significant.

## Results

### Cur20 Showed Higher Hydrolytic Stability Than Curcumin

The results showed that curcumin was easy to decompose in aqueous solvents, and the decomposition rate of Cur20 (1.36 ± 0.17%) was significantly lower than that of curcumin (30.59 ± 1.09%).

### Cur20 Restored the Formation of ISV in Injured Zebrafish Model

**FIGURE 3:**
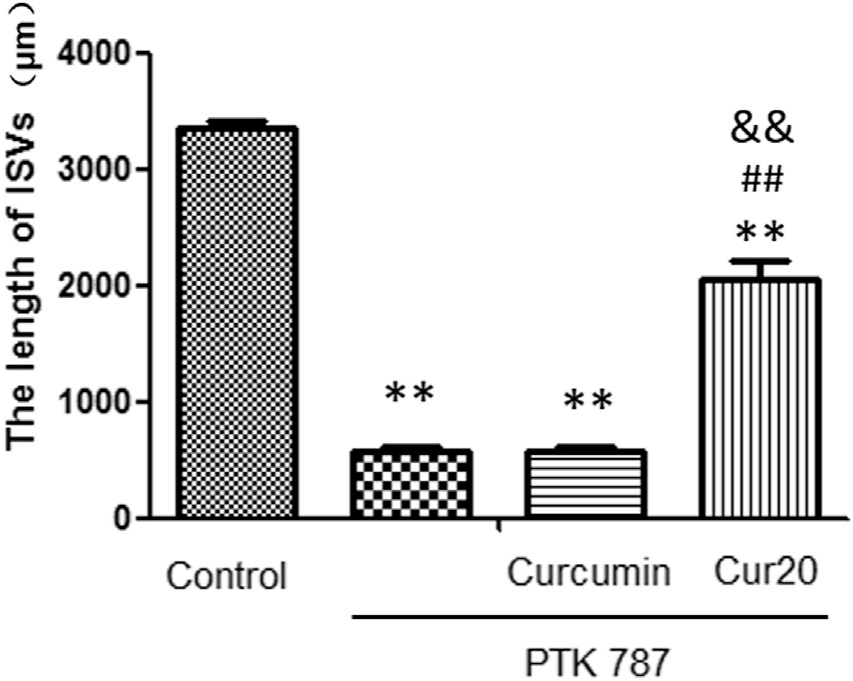
Cur20 restored the formation of intersegmental vessels (ISV) in angiogenesis inhibition model of zebrafish. After 20 hpf (hours post fertilization), zebrafish embryos were treated with drugs for 24 h, and the effect of drugs on the ISVs of zebrafish was observed and calculated using Image Pro Plus software. The experimental data was expressed by mean ± SD, *n* = 16. **, *p* ＜ 0.01 vs. control group; ##*p*＜0.01 vs model group (PTK787 group); and *p*＜0.01 vs Curcumin group.

Zebrafish, a model animal, is 87% homologous with human in gene level, which is suitable for pharmacological research of various major human diseases (Zou et al., 2017). In this study, angiogenesis inhibition model induced by PTK787 (specific VEGF receptor inhibitor) was applied to evaluated the angiogenesis effect of Cur20. Results showed that the length of ISV was significantly decreased by PTK787 treatment. Curcumin had no significant effect on the angiogenesis of the model, while Cur20 showed a significant block on the inhibition provided by PTK787 on the growth of the ISV of zebrafish (Figure 3 and Supplementary Figure S3). It suggests the potential pro-angiogenesis effect of Cur20 in pathological state.

### Cur20 Improved the Behavioral Function of the rUCCAO Mice

**FIGURE 4:**
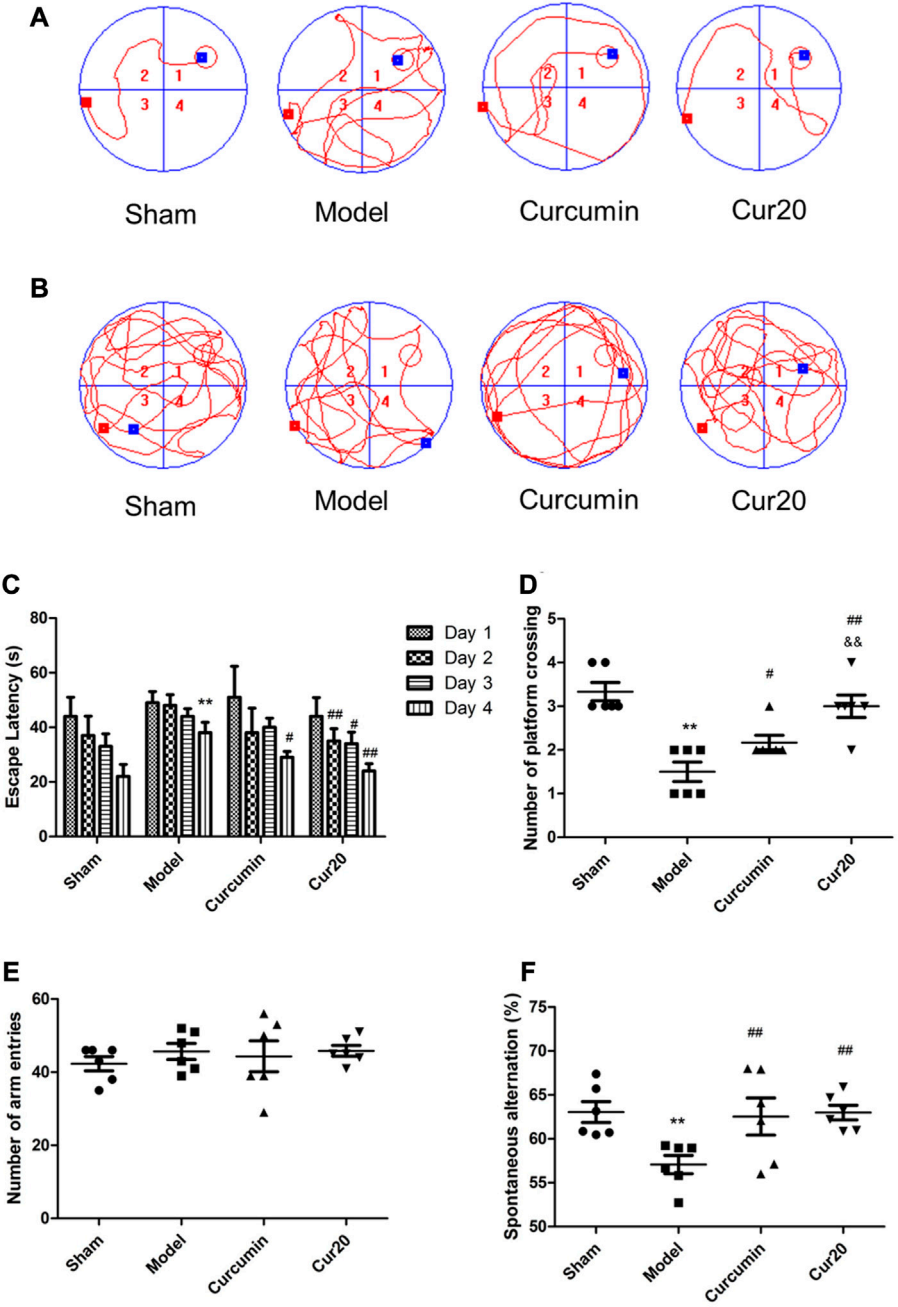
Effect of Cur20 on the learning memory **(A–D)** and working memory **(E, F)** of Vascular Dementia model. a, Representative pathways on the fourth day of navigation test; **B**, Representative swim paths on the fifth day of probe test; **C**, Escape latencies of mice during acquisition phase; **D**, Number of platform crossings of mice within 90s in probe test. **E**, The number of mice enter the three arms in the Y maze; **F**, Spontaneous alternation rate of mice. **p < 0.01 vs. control group；##*p* < 0.01 vs. Model group; &&*p* < 0.01 vs. Curcumin group.

Morris water maze is conventionally used to measure cognitive function. Figures 4A,B showed the representative pathways of navigation test and probe test of each group, respectively. The path length of mice from day1 to day4 were record according to previous research method (Bromley-Brits et al., 2011) and shown in Supplementary Figure S4. According to Figure 4C, the escape latency of the VaD group was significantly longer than that of the sham group on day 4 (*p* < 0.01), suggesting that the spatial learning ability of mice in model group was impaired. After treatment with Cur20, the escape latency on day 2, 3, 4 was decreased. In the probe trails, obvious enhancement of the times of platform crossing (Figure 4D) was found in Cur20-treated group, which was even more than that of curcumin group, indicating the recovery of cognitive function by Cur20. This result was further supported by the Y-maze assessment, in which working memory was tested. Compared with model mice, the spontaneous changes were significantly increased in Cur20 group, without significant difference of the arm entries (Figures 4E,F).

### Cur20 ameliorated the Oxidative Injuries and Morphological Damage in rUCCAO Mice

**FIGURE 5:**
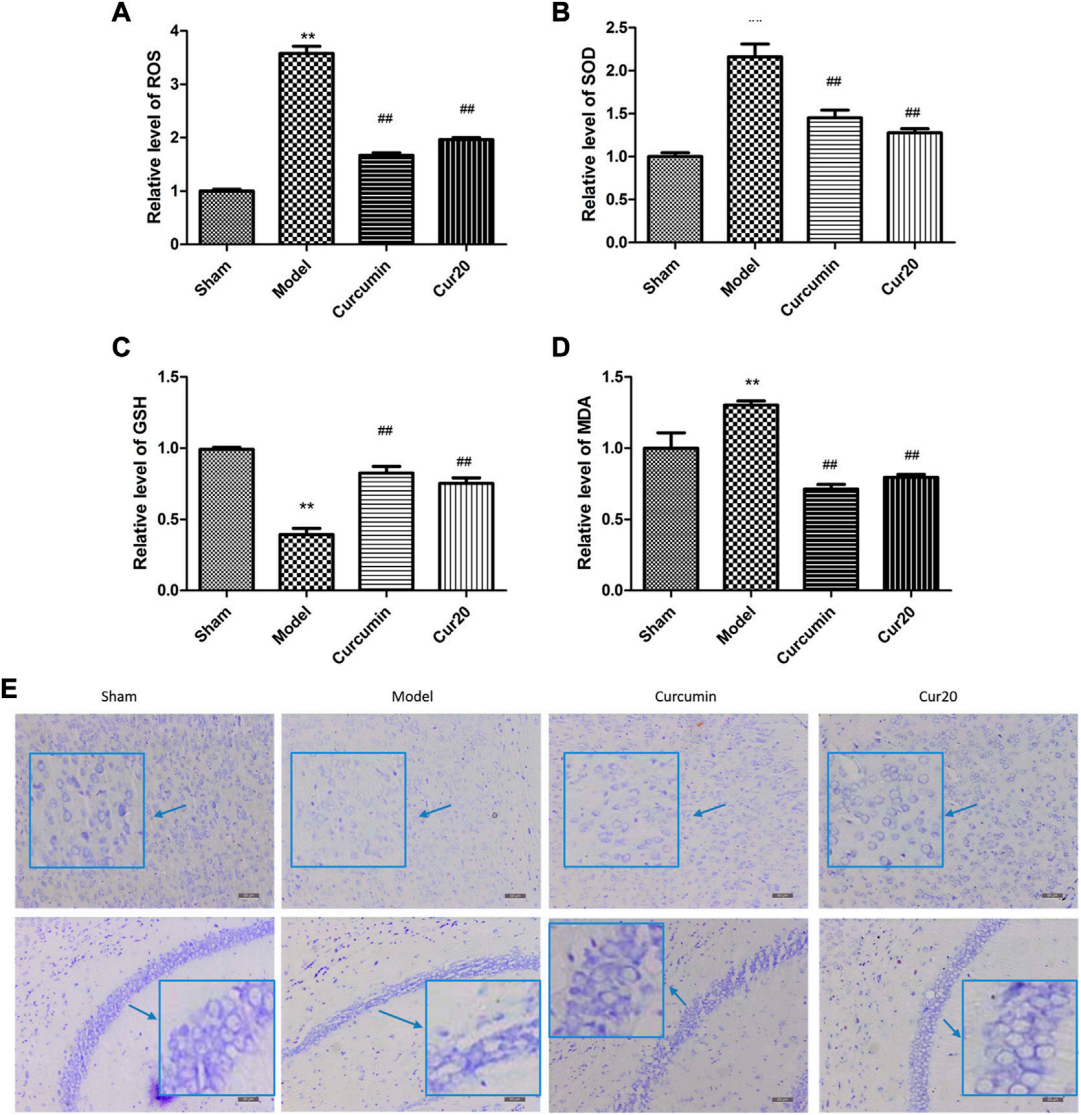
Cur20 exerted antioxidant effect and prevented neuron loss in cerebral cortex and hippocampus. Relative content of ROS **(A)**, SOD **(B)**, GSH **(C),** and MDA **(D)** in brain homogenate. **E**, Nissl stain reveal the effect of Cur20 on the morphology improvement in cortex and hippocampus in animal models of vascular dementia (200×). **p < 0.01 vs. Sham group; ##*p* < 0.01 vs. Model group.

Compared with the sham group, the ROS level increased significantly in the model group (Figure 5A). The enhanced ROS was depressed significantly by the curcumin and Cur20 treatment (*p* < 0.01). Accordingly, as the final product of peroxidation reaction between free radicals and lipids, MDA content was obviously reduced by treatment of curcumin and Cur20 after modeling (Figure 5D). Moreover, as shown in Figure 5B and c, the SOD and GSH content in the model groups both decreased sharply compared with the sham group, while curcumin and cur20 could restore the level significantly. These indicates the similar antioxidant effect of Cur20 and curcumin. As the result, the impaired neurons by chronic ischemia were decreased by drug treatment in cerebral cortex and hippocampus area. As shown in Figure 5E, neurons of the sham group were arranged in order without obvious Nissl body loss, while the neurons of the model group showed scattered arrangement, and the cell number significantly decreased. After treatment with curcumin and Cur20, the cell apoptosis decreased and Nissl body was recovered.

### Cur20 Treatment Activated HIF-1α/VEGF/TFEB Pathway-Related Angiogenesis in Mice With VaD

**FIGURE 6:**
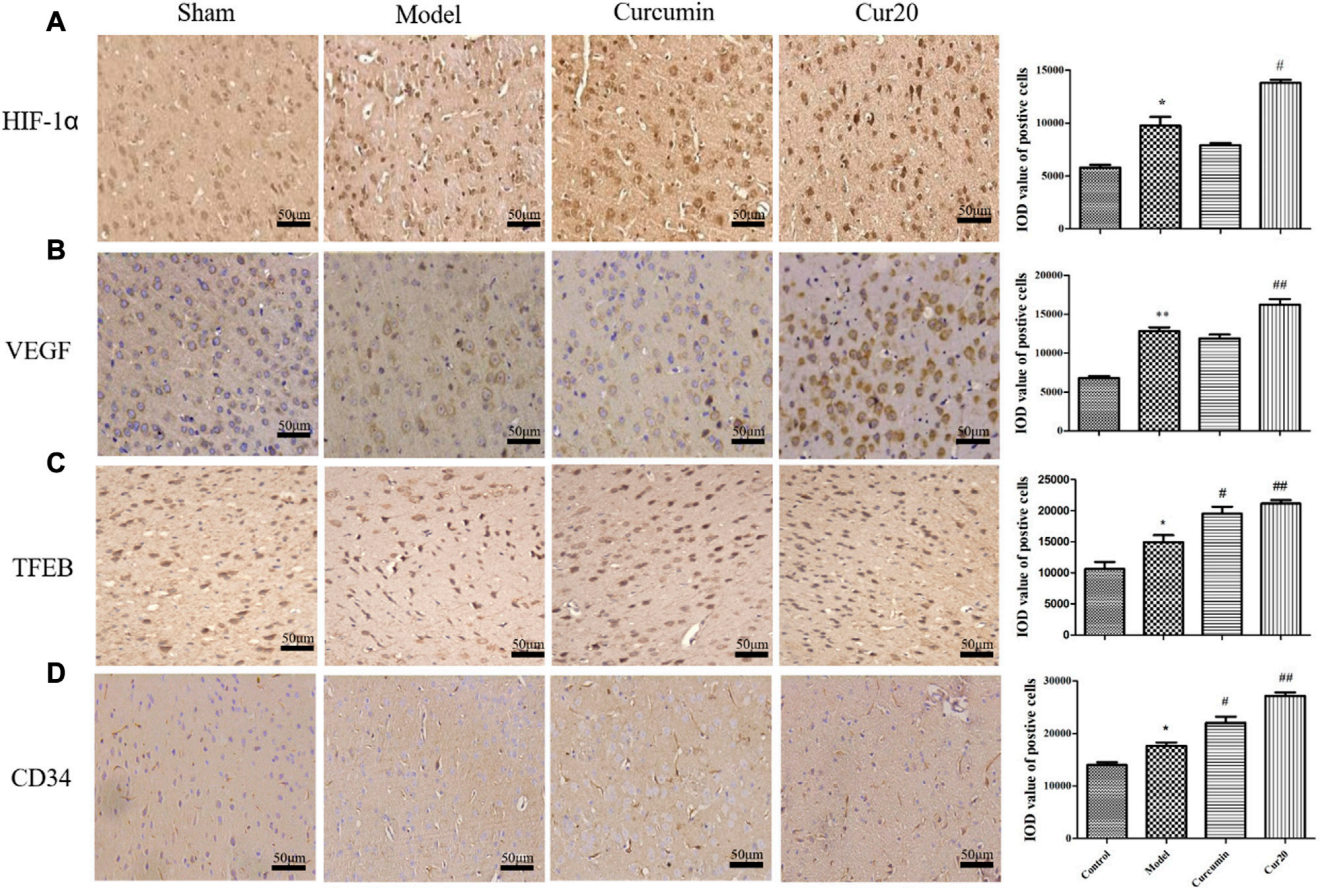
Expression of HIF-1 *a* **(A)**, VEGF **(B)**, TFEB **(C)**, and CD34 **(D)** in cerebral cortex analyzed by immunohistochemistry. *p < 0.05 vs. sham group, **p < 0.01 vs. sham group; ^#^ *p* < 0.05 vs. Model group, ##*p* < 0.01 vs. Model group.

According to immunohistochemistry analysis, the protein level of HIF-1α, VEGF, TFEB, and CD34 in the VaD group was significantly increased compared with the sham group (Figure 6), suggesting the compensation effect of angiogenesis under chronic ischemia. The curcumin group only showed much higher level of TFEB, and CD34 compared with the model group, whereas the protein levels of HIF-1α, VEGF, TFEB, and CD34 of the Cur20-treated mice were all remarkably increased in comparison with the VaD group, suggesting that Cur20 could activate HIF-1α/VEGF/TFEB-mediated angiogenesis in VaD.

### Cur20 Showed Protective Effect on OGD-Treated rBMECs

**FIGURE 7:**
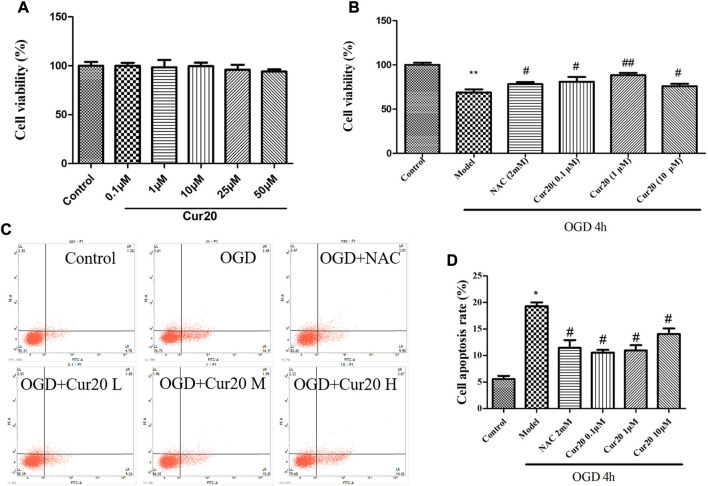
Cur20 showed protective effect on OGD-treated rBMECs without cytotoxicity. **A**, Cur20 treatment per se showed no cytotoxicity on rBMECs. **B**, Cur20 reversed OGD-induced cell damage by MTT assay. **C–D**, Flow Cytometry assay was utilized for cell apoptosis test. L, M, or H represents the concentration of Cur20 as 0.1, 1, 10 μM, respectively. The experimental data was expressed by mean ± SD, *n* = 3.*p < 0.05 vs. control group, **p < 0.01 vs. control group, #*p* < 0.05 vs. Model group, ##*p* < 0.01 vs. Model group.

As shown in Figure 7A, treatment with Cur20 under 50 μM for 48 h did not show any cytotoxicity in the rBMECs (Data of 24 and 72 h shown in Supplementary Figure S5). Antioxidant NAC was applied to reveal whether the antioxidant ability of Cur20 is vital for its protection effect. According to Figure 7B, pretreatment of 0.1 μM Cur20 showed similar protective effect on OGD-treated cells as NAC did, while 1 μM Cur20 showed much better effect. However, increase the concentration to 10 μM did not show higher survival rate. The situation was similar when 25 and 50 μM Cur20 was applied (Data not shown). Moreover, apoptosis analysis further revealed that 19.31 ± 0.67% cells were induced into apoptosis with OGD treatment, while 2 mM NAC and 0.1, 1, 10 μM Cur20 significantly reversed the apoptosis rate to 11.45 ± 0.75%, 10.54 ± 0.46%, 10.97 ± 0.37%, and 14.05 ± 0.31%, respectively.

### Cur20 Facilitated Proliferationin OGD-Treated rBMECs With Moderate Depression of ROS

**FIGURE 8:**
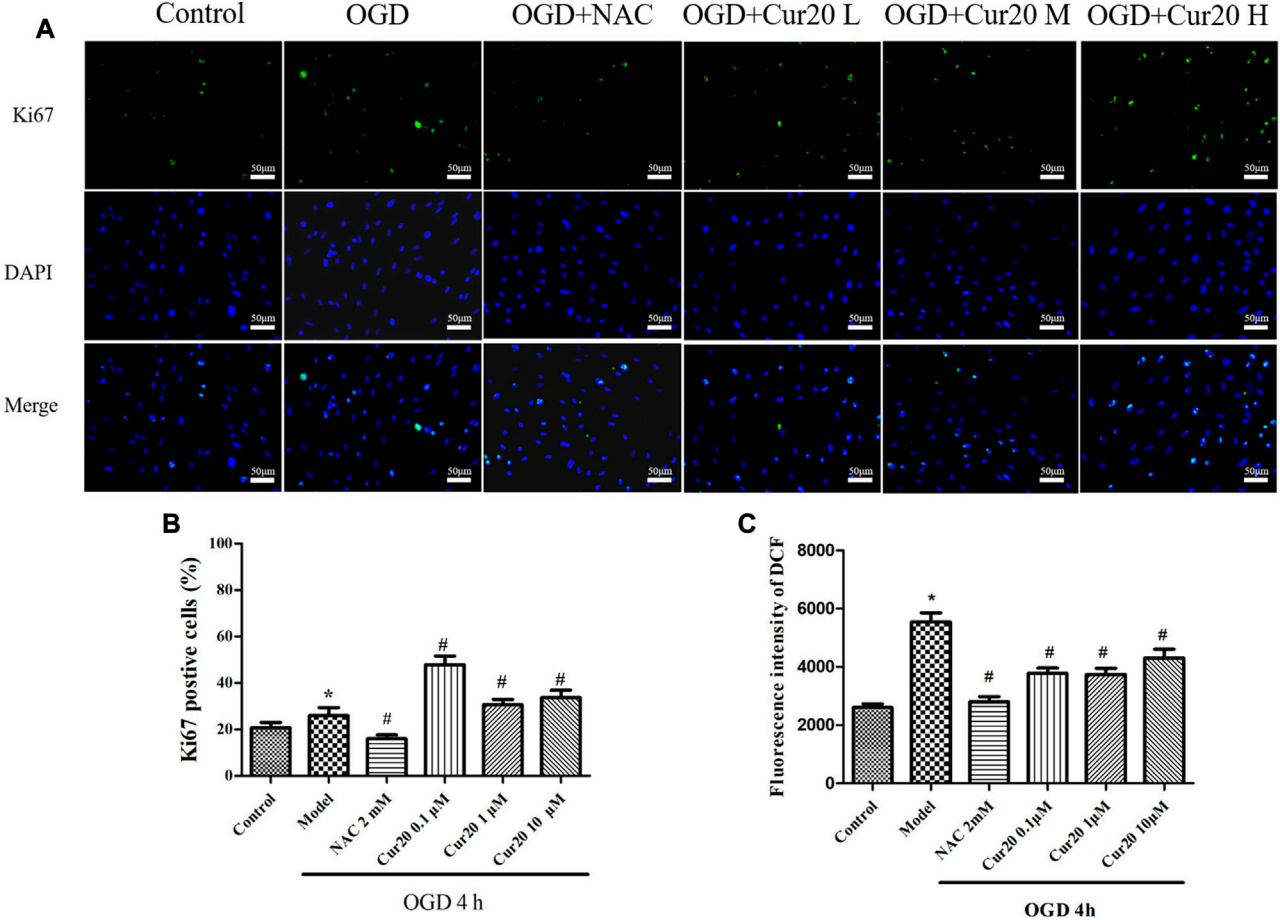
Effect of Cur20 on the proliferation of rBMECs after treatment with OGD for 4 h and cultivated for another 24 h **A–B**, Immunofluorescence was utilized to observe the positive stain of Ki67 (200×). **C**, DCFH-DA was used to detect the intracellular ROS level. The experimental data was expressed by mean ± SD, *n* = 3. *p < 0.05 vs. Control group, #*p* < 0.05 vs. Model group.

The expression of Ki67, a cell proliferation marker (Wan et al., 2019), was detected by immunofluorescence method, and the rate of proliferation of each group was measured and shown in Figure 8. Compared with the control group, the rate of Ki67 positive cells in the model group increased after 4 h of hypoxia treatment with 24 h further culture. Compared with the model group, the rate significantly decreased after pretreatment with 2 mM NAC for 2 h, but increased when pretreated with Cur20. We further analyzed the ROS content in the rBMECs. The result showed that NAC significantly depressed the intracellular ROS content induced by OGD, while Cur20 only moderately decreased the ROS level in the damaged rBMECs.

### The Effect of Cur20 on Angiogenesis-Related Pathway

**FIGURE 9:**
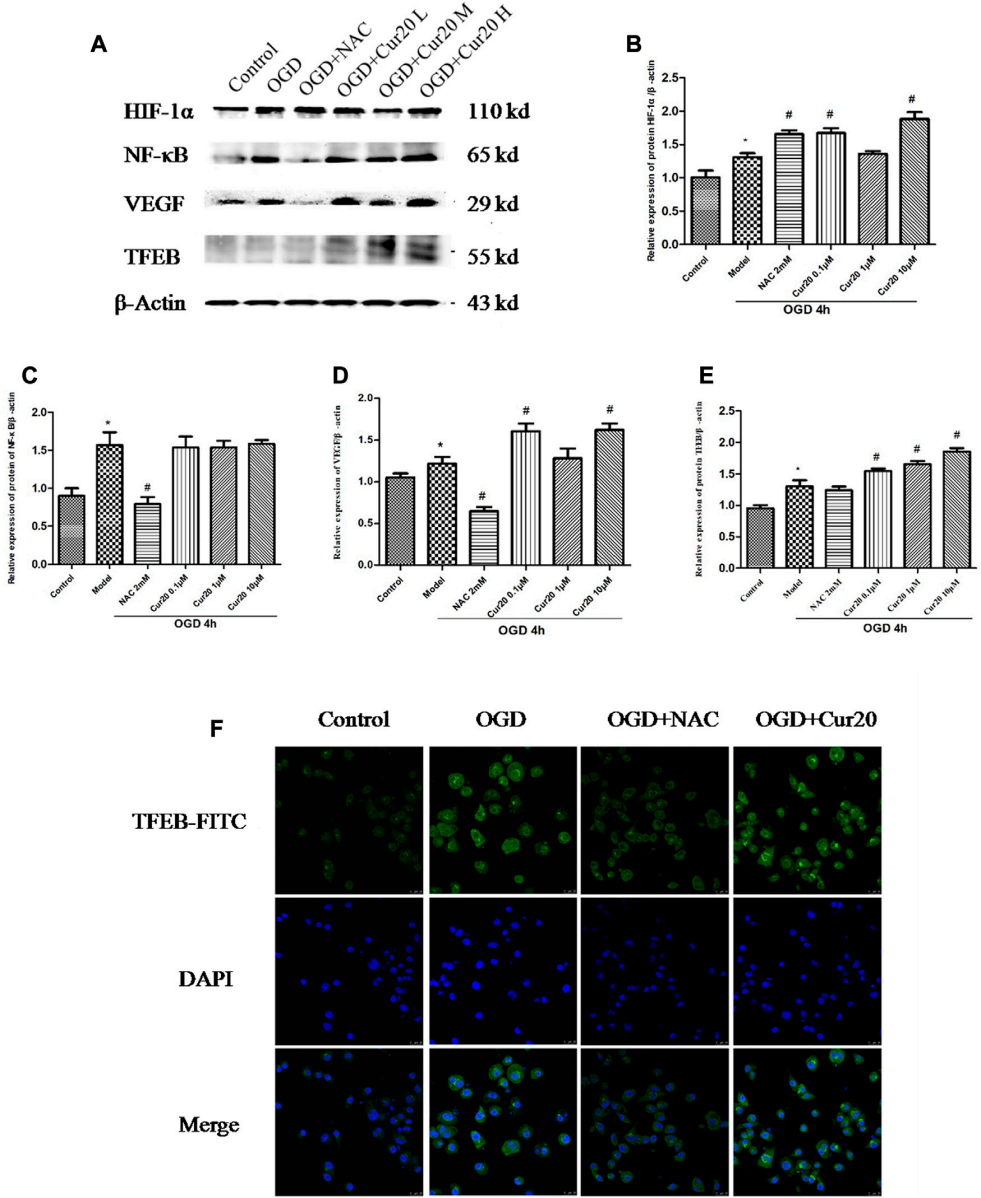
Effect of Cur20 on the HIF-1α/VEGF/TFEB pathway. **A–E**, The protein levels of HIF-1α, NF-κB, VEGF, and TFEB analyzed by western blot. **F**, The content and translocation of TFEB in nuclei observed by confocal microscopy with immunofluorescence. rBMECs were pretreated with Cur20 for 2 h then treated with OGD for 4 h. L, M, or H represents the concentration of Cur20 as 0.1, 1, 10 μM, respectively. In the immunofluorescence experiment, the concentration of Cur20 was 10 μM. The experimental data was expressed by mean ± SD, *n* = 3.*p < 0.05 vs. control group, ^#^ *p* < 0.05 vs. Model group.

As shown in Figure 9, OGD treatment led to the protein level of HIF-1α, NF-κB, VEGF, and TFEB increased. Cur20 further enhanced the expressions of HIF-1α, VEGF, and TFEB, especially when treatment concentration was 0.1 and 10 μM. NAC treatment only facilitated the expression of HIF-1α, while NF-κB, VEGF, and TFEB expression was inhibited significantly. The translocation and the content of TFEB evaluated by immunofluorescence in Figure 9F shows the similar trend that Cur20 increased TFEB activation but NAC did not.

## Discussion

Because curcumin has good biological activity and poor hydrolysis stability, we intend to construct curcumin analogues with high hydrolysis stability through structural modification. Among those analogues, Cur20 has been shown to have similar antioxidant activity and much higher stability than curcumin: the total antioxidant capacity of curcumin and Cur20 were 1.39 ± 0.16 and 1.35 ± 0.14 times of that of vitamin E, respectively (determined by Antioxidant capacity test kit, Beyotime Institute of Biotecnology, Nantong, China. Data not shown). Curcumin has been reported to have neuroprotective effects due to its antioxidant and anti-inflammatory effects (Mhillaj et al., 2019). However, curcumin showed bidirectional action on angiogenesis in different pathological microenvironment (Wang and Chen, 2019). According to the screen experiment on zebrafish, Cur20 showed much better effect on angiogenesis than curcumin. Further experiments on rats also confirmed that Cur20 had a significant effect on angiogenesis *in vivo*, suggesting it has more advantages for ischemic diseases like VaD.

On rUCCAO mice, Cur20 treatment not only protected more neurons in the cerebral cortex and hippocampus, but also improved the overall behavioral ability, even better than curcumin in some indexes. The major reason may be the ameliorated oxidative injuries, which was verified by results of ROS, MDA, SOD, and GSH *in vivo*. Although there are a series of pathophysiological changes after cerebral ischemia, oxidative stress is still one of the most serious strikes among Ca^2+^ overload, excitatory amino acid overexpression, inflammatory response and so on (Zhao et al., 2019; Luca et al., 2015). During cerebral ischemia, mitochondrial function is abnormal with free radicals accumulation, which leads to lipid, protein, and DNA damage, causing apoptosis and irreversible necrosis. Therefore, we speculate that curcumin and Cur20-played protective role mainly through antioxidation.

However, it is worthy to note that solely antioxidant NAC showed less protective and proliferative effect *in vitro* experiments. It means that complete elimination of ROS may not be the best treatment for VaD, while the appropriate antioxidant effect of Cur20 should play a better protective role. As known, ROS are byproducts of normal oxidative metabolism of cells that have many physiological activities like activation in proliferation and so on (Yu et al., 2019; Song et al., 2014). Further experiments revealed that while Cur20 removed a large amount of ROS, it also left some ROS content after drug treatment. In addition to substantially reducing oxidative damage, certain content of ROS may play a positive role in the activation of the intracellular antioxidant system, proliferation system and cellular renew system. As we reviewed (Cen et al., 2013), although antioxidants could significantly reduce the injury caused by oxidative stress, their low effectiveness and side effects suggest we should pay more attention to the positive roles of ROS in drug application (Singh et al., 2017).

**FIGURE 10:**
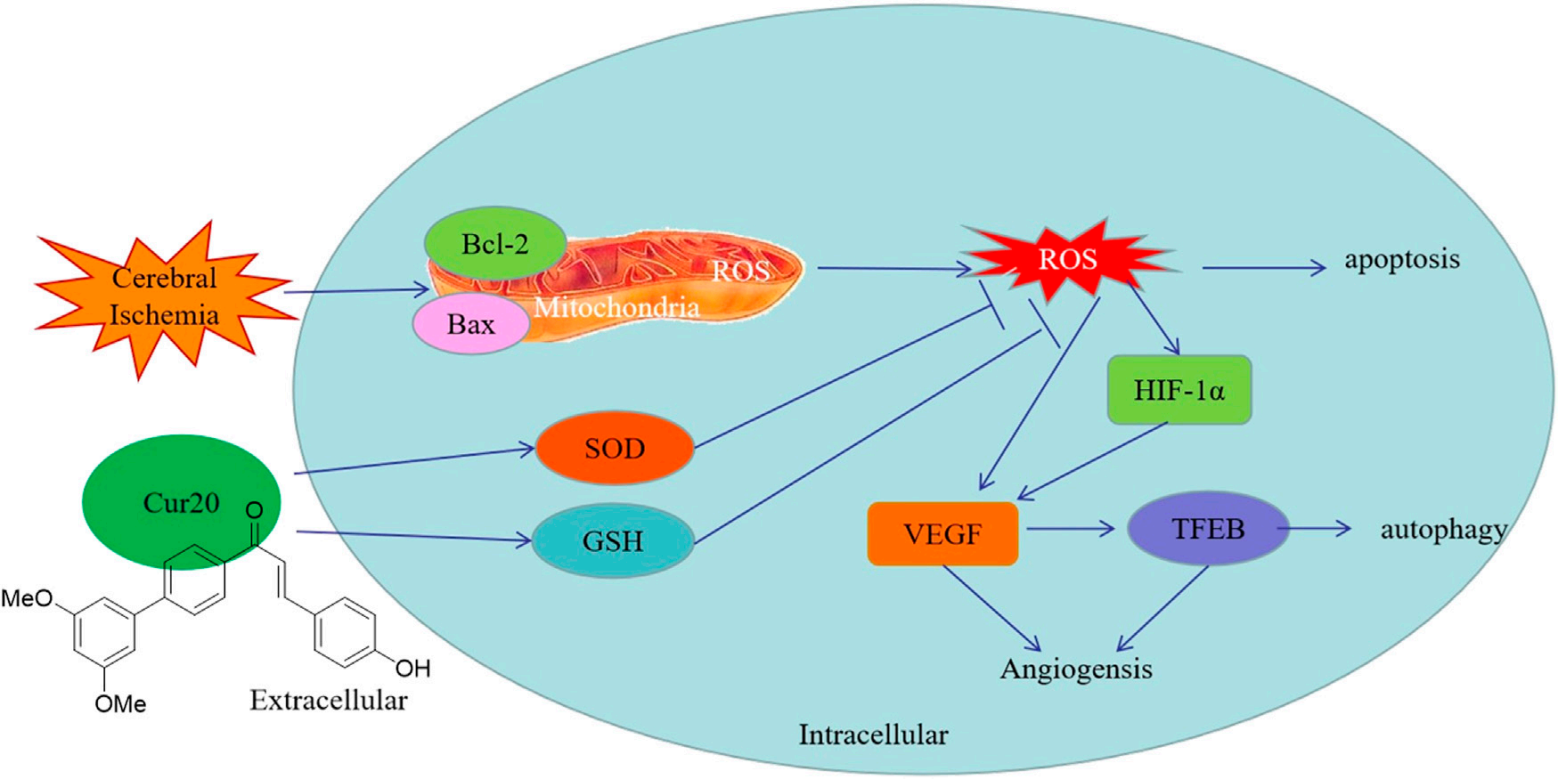
Possible protection mechanism of Cur20 against ischemic injury in rBMECs.

Studies have shown that ROS are not only the core factor in ischemic brain injury, but also involved in the tissue repair during convalescence (Chan et al., 2016). After ischemia, certain amount of ROS is needed in the recovery processes including proliferation and differentiation. After a few minutes to several hours of cerebral ischemia, ROS were produced in large quantities, and continued to rise for a few days until it gradually returned to normal around 20 days later (Yi et al., 2012). Since the cell proliferation in the brain is consistent with the generation trend of ROS, it is believed that ROS are involved in neurogenesis in the late stage of stroke. More importantly, different ROS concentrations activate different signal transduction pathways and produce corresponding physiological changes (Xiaonan et al., 2018). Further studies have shown that ROS produced by ischemia can activate hypoxia inducible factor (HIF) and downstream pathway, in which the changes of Notch pathway, Wnt/β-Catenin pathway, and hypoxia induced growth factors (such as fibroblast growth factor-2, epidermal growth factor, cyclin dependent kinase inhibitor p27) are involved in neural stem cell differentiation, neurogenesis, neuronal migration (Sutton et al.). Therefore, ROS are considered to participate in the tissue repair in the late stage of ischemia. Consistently, compared with NAC, Cur20 significantly reduced but did not completely eliminate ROS, resulting in a greatly increased HIF-1α regulation expression with enhanced rBMECs proliferation. Since VEGF is the most important angiogenic factor and the target gene of HIF-1α, we also tested the expression of VEGF. As a promoter of endothelial cell proliferation and migration, VEGF was found to be increased by Cur20, while the increase of rBMECs proliferation was also confirmed. In addition, VEGF can also protect neurons from ischemic injury and improve synaptic plasticity, which may be related to the effect of VEGF on TFEB. TFEB, which is considered as the key regulator of lysosomal autophagy pathway, with the function of regulating autophagy and enhancing lysosomal biological activity (Neill et al., 2017). Studies have shown that TFEB can improve neurodegenerative diseases, such as Huntington’s disease and Parkinson’s disease. Fan et al. have found that VEGF can enhance TFEB activity, and thus regulate angiogenesis and promotes cell survival, thereby reducing brain injury after ischemia (Fan et al., 2018). As we have shown in this study, the possible protection mechanism of Cur20 against ischemic injury in rBMECs may also involve the activation of angiogenesis by HIF-1α/VEGF/TFEB (Figure 10).

Overall, since many kinds of neurodegeneration diseases share vascular risk factors as the common pathological mechanism (Kamal et al., 2016), Cur20 may be promising for further development.

## Data Availability

The original contributions presented in the study are included in the article/Supplementary Material, further inquiries can be directed to the corresponding authors.
