# Supplementary material for: Curcumin Derivative Cur20 Attenuated Cerebral Ischemic Injury by Antioxidant Effect and HIF-1α/VEGF/TFEB-Activated Angiogenesis
Source: Front Pharmacol. 2021 Apr 15;12:648107. doi: 10.3389/fphar.2021.648107 (PMC8082391; doi:10.3389/fphar.2021.648107)
Supplement: Supplementary file 1 [file datasheet1.docx]

Supplementary material

1. Synthesis

1.1. Materials and methods

All reactions were carried out under anhydrous conditions, using flame-dried glassware under an nitrogen atmosphere. CH_2_Cl_2_ was distilled using CaH_2_. Other reagents were used directly without any treatment as soon as obtained from commercial suppliers. TLC was performed on silica gel plates with UV and p‑anisaldehyde or KMnO4 stain visualization. Purification through flash chromatography was performed on silica gel (230−400 mesh). ^1^ H-NMR spectra were recorded at 400 MHz in CH_3_OH-*d_4_*, CDCl_3_, or CD_3_OD, and data are reported as follows: chemical shift in ppm from tetramethylsilane as an internal standard, multiplicity (s = singlet, d = doublet, t = triplet, q = quartet, quint = quintuplet, m = multiplet or overlap of nonequivalent resonances, br = broad), integration. ^13^C-NMR spectra were recorded at 100 MHz in CDCl_3_ or CD_3_OD, and data are reported as follows: chemical shift in ppm from tetramethylsilane with the solvent as an internal standard (CDCl_3_, 77.16 ppm or CD_3_OD, 49.00 ppm). Mass spectra were realized with a gas chromatograph−mass spectrometer by electronic impact. High resolution mass spectra (HRMS) were performed with an orbitrap mass analyzer by electrospray ionization.

The curcumin analogue Cur20 (1 - (3 ', 5 '- dimethoxy - [1, 1' - - yl) biphenyl] - 4-3 - (4 - hydroxyphenyl) prop - 2 - en - 1 - one ) was prepared starting from *p*-hydroxybenzaldehyde through five steps.

1.2. General procedure for the preparation of 4-((tetrahydro-2H-pyran-2-yl)oxy)benzaldehyde (**compound** **1**)

To a 100 ml round bottom flask containing 20.0 mL dry dichloromethane was added 1.40 g *p*-hydroxybenzaldehyde (11.46 mmol), followed by the addition of 2.0 mL 3, 4-dihydro-2H-pyran. After a homogeneous solution as formed, a solution of camphor sulfonic acid (CSA) (5.0 mg dissolved in 5.0 mL dry dichloromethane) was added and the mixture solution was then stirred at room temperature overnight. After the solvent was evaporated, purification of the residue through silica gel chromatography using petroleum ether: ethyl acetate (V:V= 4:1) as eluent afforded 2.27 g **compound** **1** as a yellowish oil in yield of 96%.

1.3 General procedure for the preparation of (E)-1-(4-bromophenyl)-3-(4-((tetrahydro-2H-pyran-2-yl)oxy)phenyl)prop-2-en-1-one (**compound** **2**)

Compound **1** (0.413 g, 2 mmol) was dissolved in 5.0 mL of methanol and then cooled to 0^o^C, followed by the addition of [4'-bromoacetophenone](http://www.baidu.com/link?url=IUTi0wHJ2p4L9-li9oyTASEN1wnyIGPNZotN8f5c9uC1GTcI178WRbrA_9ddy_vM571L69nj3oNthriQOIA3YSgaZ_st8NHG7DjlQ04loMG) (0.398 g, 2 mmol). After the solution was stirred at 0^o^C for 5 minutes, 5.0 mL NaOH solution (5.0 M) was added dropwise. The reaction proceeded at 0^o^C for 10 min and then the temperature gradually rose to room temperature. After the starting materials were consumed completely, the solution was extracted with dichloromethane extraction (50 mL x3).The organic solution was combined and evaporated under reduced pressure. Purification of the residue through silica gel column chromatographyemploying petroleum ether, ethyl acetate (V: V = 10:1) as eluent afforded 0.489 g of the desired product, **compound** **2** in yield of 63%.

1.4 General procedure for the preparation of 2-(3,5-dimethoxyphenyl)-4,4,5,5-tetramethyl-1,3,2-dioxaborolane (**compound** **3**)

Pinacol borate (B_2_pin_2_) (1.524 g, 6 mmol), Ir (OMe) (COD) (0.133 g, 0.2 mmol) and 4,4-*Di*-*tert*-butyl bipyridine (dtbpy) (0.107 g, 0.4 mmol) were dissolved in 15 mL of cyclohexane under N_2_ atmosphere at room temperature, followed by the addition of 1,3-dimethoxy benzene (1.22 mL, 8 mmol). After the reaction proceeded for 10 hours, the solvent was evaporated under reduced pressure, and the residue was purified through column chromatography employing petroleum ether, ethyl acetate (V: V = 5 to 1) as eluent, affording 1.445 g of **compound 3** in yield of 68%.

1.5 General procedure for the preparation of (E)-1-(3',5'-dimethoxy-[1,1'-biphenyl]-4-yl)-3-(4-((tetrahydro-2H-pyran-2-yl)oxy)phenyl)prop-2-en-1-one (**compound** **4**)

**Compound** **2** (0.387 g, 1.0 mmol), **compound** **3** (0.317 g, 1.2 mmol), Cs_2_CO_3_ (0.346 mg, 1.05 mmol), and Tetrakis(triphenylphosphine)palladium(0)(Pd(PPh_3_)_4_) (34.7 mg, 0.03 mmol) were added into a microwave tube, followed by the addition of add 1.0 mL of distilled water and 5.0 mL dimethyl formamide/ethanol (V: V = 4:1). After the mixture was treated in a microwave at 100^o^C for 1 h, the resultant solution was extracted with methylene chloride (50 mL x3).Then the combined organic solution was washed with distilled water to remove the dimethyl formamide (DMF) and dried with anhydrous magnesium sulfate. After filtration, the solution was condensed under reduced pressure and the residue was purified through column chromatography employing petroleum ether: ethyl acetate (V:V=8:1) as eluent to afford 0.360 g of compound **4** in yield of 81%.

1.6 General procedure for the preparation of (E)-1-(3',5'-dimethoxy-[1,1'-biphenyl]-4-yl)-3-(4-hydroxyphenyl)prop-2-en-1-one (**compound** 5, Cur20)

Compound **4** (0.356 g, 0.8 mmol) was dissolved in1.0 mL dimethyl sulfoxide (DMSO), followed by the addition of 10.0 mL methylene chloride and *p*-Toluene sulfonic acid monohydrate (1.52 g, 8 mmol). After the reaction proceeded at room temeperature for 10 h, the mixture solution was washed with water to remove the DMSO.The organic phase was dried with MgSO_4_, filtered, and condensed under reduced pressure prior to the purification of the residue through column chromatography employing petroleum ether and ethyl acetate (V: V = 2:1) as eluent to afford compound **5** as a yellow solid (0.236 g) in yield of 82 %. ^1^H NMR (400 MHz, CH_3_OH-d_4_) δ 8.16-8.10 (m, 2H), 7.81-7.74 (m, 3H), 7.67-7.58 (m, 3H), 6.88- 6.84 (m, 2H), 6.82 (d, *J* = 2.3 Hz, 2H), 6.54 (t, *J* = 2.2 Hz, 1H), 3.85 (s, 6H); ^13^C NMR (100 MHz, CH_3_OH-d_4_) δ 190.66, 161.43, 160.47, 145.51, 145.42, 141.88, 137.22, 130.51, 128.72, 126.94, 126.28, 118.21, 115.60, 105.03, 99.65, 54.50; HRMS (DART-TOF) calculated for C_23_H_20_O_4_ [M+Na]^+^ m/z 383.1254.

The structure of compounds 1-5 is shown in Fig.S1 below:

Fig.S1 The structures of **compounds 1-5**

2. Chemistry

The synthetic route of curcumin analogue Cur20 is outlined in Fig.S2. 3, p-hydroxybenzaldehyde was converted to **1** in yield of 96% in the presence of 4-dihydro-2H-pyran and camphor sulfonic acid (CSA), which then reacted with [4'-bromoacetophenone](http://www.baidu.com/link?url=IUTi0wHJ2p4L9-li9oyTASEN1wnyIGPNZotN8f5c9uC1GTcI178WRbrA_9ddy_vM571L69nj3oNthriQOIA3YSgaZ_st8NHG7DjlQ04loMG)to form **2** in yield of 63%. The reaction of pinacol borate (B2pin2) with 4, 4-di-tert-butyl bipyridine (dtbpy) using Ir (OMe) (COD) as catalyst afforded compound 3 in yield of 68%. Subsequently, **2** reacted with **3** in presence of Tetrakis(triphenylphosphine)palladium(0)(Pd(PPh_3_)_4_) and Cs_2_CO_3_ to produce **4** in yield of 81%, followed by the removal of the protecting group employing TsOH to form **5** quantitively (Cur20).

Fig.S2 Synthesis of curcumin analogue Cur20. Reagents and conditions: i. *p*-hydroxybenzaldehyde, 3, 4-Dihydro-2H-pyran, DCM, CSA, r.t., overnight; ii. [4'-Bromoacetophenone,](http://www.baidu.com/link?url=IUTi0wHJ2p4L9-li9oyTASEN1wnyIGPNZotN8f5c9uC1GTcI178WRbrA_9ddy_vM571L69nj3oNthriQOIA3YSgaZ_st8NHG7DjlQ04loMG)MeOH, 0^o^C-r.t.; iii. Pinacol borate (B_2_pin_2_), 4,4-*Di*-*tert*-butyl bipyridine (dtbpy), cyclohexane, N_2_, 10 h; iv. Pd(PPh_3_)_4_, Cs_2_CO_3_, H_2_O_2_:DMF:EtOH (1:1:4, v/v), 100^o^C, 1 h; v. TsOH, DMSO:DCM (1:10 v/v), r.t., 10 h.


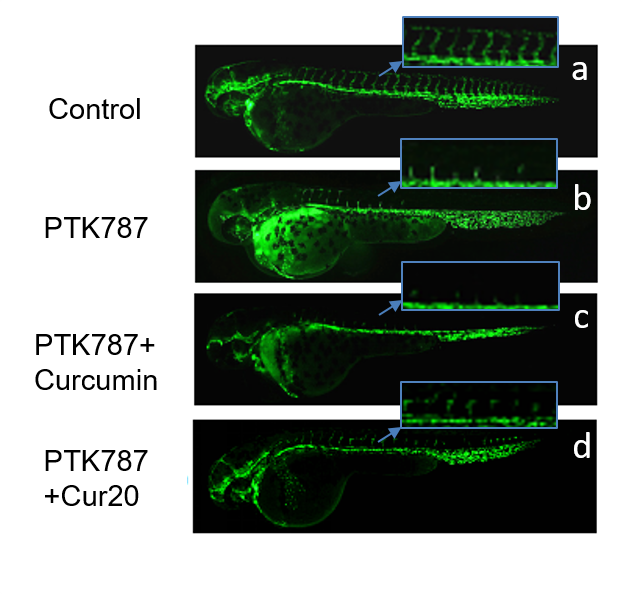


Fig.S3 Cur20 restored the formation of intersegmental vessels (ISV) in angiogenesis inhibition model of zebrafish. a-d: after 20 hpf (hours post fertilization), zebrafish embryos were treated with drugs for 24 hours, and the effect of drugs on the ISVs of zebrafish was observed by fluorescence microscope (4×). (a) Control (0.1% DMSO), (b)-(d) drug administration groups: PTK787 (0.2 μg/ml) treatment group, curcumin (50 μM) + PTK787 (0.2 μg/ml) treatment group, cur20 (50 μmol/L) + PTK787 (0.2 μg/ml) treatment group, respectively.


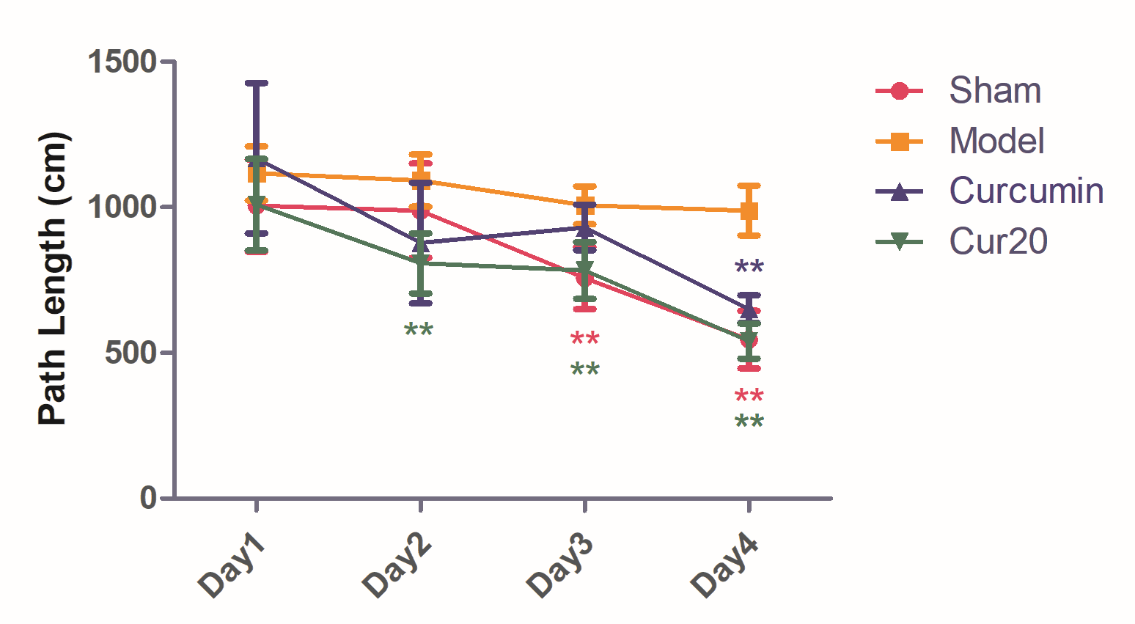


Fig.S4 The Cur20-treated rUCCAO mice had a shorter swimming length before escaping onto the hidden platform on the 3rd and 4th day. N=6. **：p<0.01 vs. control group.


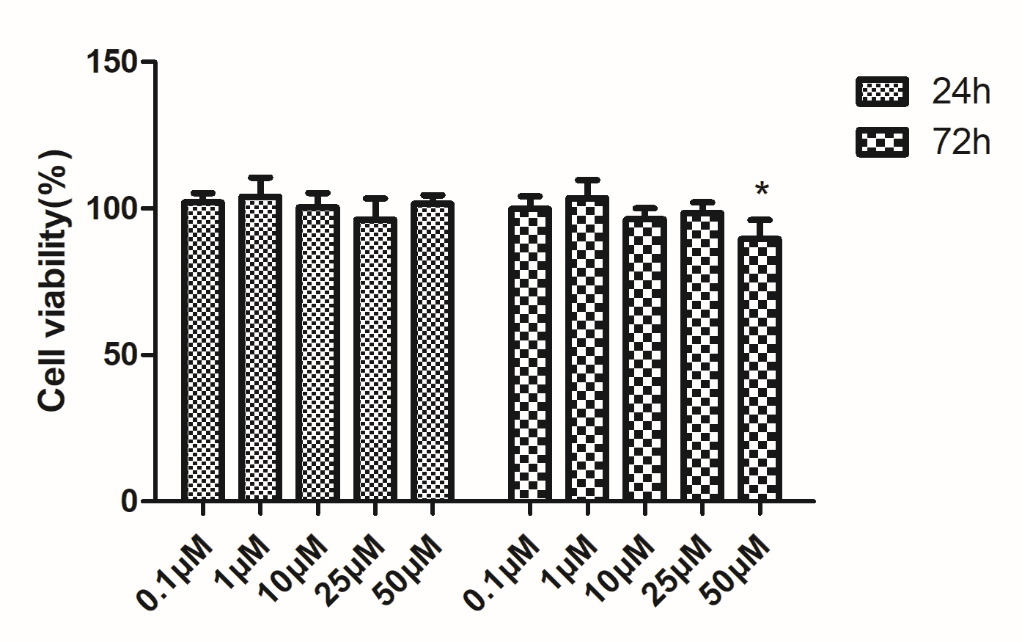


Fig.S5 Cell viability after treatment of Cur20 for 24 and 72 h on rBMECs. The experimental data was expressed by mean ± SD, n = 3.**p*<0.05 vs. control group, **p*<0.05 vs. control group.
